# Assessing of case–cohort design: a case study for breast cancer patients in Xinjiang, China

**DOI:** 10.3389/fonc.2024.1306255

**Published:** 2024-03-20

**Authors:** Mengjuan Wu, Tao Zhang, Chunjie Gao, Ting Zhao, Lei Wang, Gang Sun

**Affiliations:** ^1^ Country College of Public Health, Xinjiang Medical University, Urumqi, China; ^2^ Department of Medical Record Management, The Affiliated Cancer Hospital of Xinjiang Medical University, Urumqi Xinjiang, China; ^3^ Department of Medical Engineering and Technology, Xinjiang Medical University, Urumqi Xinjiang, China; ^4^ Xinjiang Cancer Center/ Key Laboratory of Oncology of Xinjiang Uyghur Autonomous Region, Urumqi, Xinjiang, China; ^5^ Department of Breast and Thyroid Surgery, The Affiliated Cancer Hospital of Xinjiang Medical University, Urumqi, Xinjiang, China

**Keywords:** case-cohort design, breast cancer, survival prognosis, Cox proportional hazards model, simulations study

## Abstract

**Objective:**

To assess the effectiveness and clinical value of case–cohort design and determine prognostic factors of breast cancer patients in Xinjiang on the basis of case–cohort design.

**Methods:**

The survival data with different sample characteristics were simulated by using Cox proportional risk models. To evaluate the effectiveness for the case–cohort, entire cohort, and simple random sampling design by comparing the mean, coefficient of variation, etc., of covariate parameters. Furthermore, the prognostic factors of breast cancer patients in Xinjiang were determined based on case–cohort sampling designs. The models were comprehensively evaluated by likelihood ratio test, the area under the receiver operating characteristic curve (AUC), and Akaike Information Criterion (AIC).

**Results:**

In a simulations study, the case–cohort design shows better stability and improves the estimation efficiency when the censored rate is high. In the breast cancer data, molecular subtypes, T-stage, N-stage, M-stage, types of surgery, and postoperative chemotherapy were identified as the prognostic factors of patients in Xinjiang. These models based on the different sampling designs both passed the likelihood ratio test (*p*<0.05). Moreover, the model constructed under the case–cohort design had better fitting effect (AIC=3,999.96) and better discrimination (AUC=0.807).

**Conclusion:**

Simulations study confirmed the effectiveness of case–cohort design and further determined the prognostic factors of breast cancer patients in Xinjiang based on this design, which presented the practicality of case–cohort design in actual data.

## 1 Introduction

Breast cancer with a high mortality rate is one of the most widespread malignant tumors, which seriously threatens women’s health and safety. Global Cancer Statistics 2020 pointed out that there were 2.27 million new cases of breast cancer worldwide, and approximately one in eight patients died of breast cancer in 2020 (1). Since the twenty-first century, the morbidity and mortality of female breast cancer in China have been continuously increasing (2), which would cause tremendous burden of breast cancer. Furthermore, breast cancer is highly heterogeneous, with the variety in molecular subtype, clinical stage, and other pathological features (3). The differences in tumor cell growth rate, invasion ability, and potential metastasis are strongly correlated with patients’ survival prognosis (4). Survival analysis is widely applied to investigate the relationship among survival time, survival state, and important influencing factors of breast cancer patients. For instance, Ma et al. (5) studied the serum lipid changes in breast cancer patients during neoadjuvant chemotherapy and the impact of dyslipidemia on their prognosis. Zhou et al. (6) identified the potential prognostic factors of patients with triple-negative breast cancer and built the corresponding prediction model.

In China, there are endemical variety in the morbidity and mortality of breast cancer (7). Relevant studies (8–10) showed that the current situation of breast cancer in Xinjiang is different from that in other regions, with such features as lower incidence rate, luminal breast cancer appearing more frequently, and women aged 45–55 having a higher risk of developing this disease. At present, there have been many studies evaluating the prognostic risk factors of patients with breast cancer in Xinjiang (11–14); for instance, Shan et al. (11) investigated the clinicopathological features and prognostic characteristics of patients with triple-negative breast cancer in Xinjiang, based on clinical information for 319 patients. Fu et al. (13) focused on the difference in survival and prognosis of breast cancer patients with different molecular subtypes in Xinjiang. Cao et al. (14) evaluated the association of hypoxia-inducible factor-1α and survivin with breast cancer prognosis in breast cancer patients. However, the sample size of some studies was relatively small (11, 12), and those studies were mainly focus on exploring the impact of molecular subtypes or gene expression on the prognosis of breast cancer patients (11, 13, 14). On the other hand, it is necessary to follow up a large number of research subjects over the long-term in survival analysis, which may inevitably cause certain omissions in the process of data collection. Realistically, the breast cancer patients followed up by the hospitals or cancer centers are equivalent to random sample from the overall population. Therefore, it could not totally represent the basic characteristics of the overall population to a certain extent. In particular, a previous study showed that the mortality rate of breast cancer in Xinjiang Cancer Registration Area was only approximately 8.72% (15). When the incidence of interested event in the follow-up subjects is lower, directly using the data of random samples would cause the insufficient power of statistical analysis (16). To decrease the sampling error produced by simple random sampling, Prentice (17) proposed the case–cohort design in 1986. On the basis of simple random sampling, the case–cohort design analyzes those patients who have experienced outcome events in the full cohort, which is suitable for these studies with lower incidence of disease outcomes or higher costs of covariate collections (18–20), and compared with the simple random sampling, the case–cohort design may decrease the sample error (21, 22). Yu et al. (18) separately investigated the relationship between demographic characteristics, tumor histology, and time of onset and recurrence of nephroblastoma patients, under a case–cohort design. Cai et al. (19) employed a case–cohort design to identify the influencing factors of fungal infection in patients with hematopoietic cell transplant. Particularly, the case–cohort design is widely used to analyze the factors influencing morbidity or mortality of breast cancer (20–22). For example, based on the case–cohort design, Yang et al. (20) used additive risk model to explore the major prognostic factors of patients with breast cancer. The case–cohort design was employed to evaluate the prospective associations between perfluoroalkyl substances and breast cancer risk in (21). Yao et al. (22) used case–cohort design to investigate the association of serum biomarker of vitamin D status, 25-hydroxyvitamin D values with breast cancer recurrence, and survival prognosis. It was indicated that the results based on the case–cohort design with fewer samples were similar to those based on the full cohort. The case–cohort design could be not only suitable for large cohort studies with low incidence but also availably reduce the cost and improve the efficiency. Furthermore, there may be a lack of repeatability in the analysis of actual clinical data; thus, using a case–cohort design could partly decrease the bias generated by random sampling. Therefore, it is significant to further determine the prognostic factors of breast cancer patients in Xinjiang by using a case–cohort design, which could contribute to explore patients’ clinical treatments and improve their survival probability.

Inspired by the aforementioned discussion, in this paper, we first explored the effectiveness of the case–cohort sampling design by using simulated data. To do this, we employed the Cox proportional hazards model to fit the parameters of covariates in these models under full cohort, case–cohort with different sampling proportions, and simple random sampling designs, respectively, and then, we compared these estimated values of parameters for those models (such as the mean, standard deviation, coefficient of variation, and bias). Second, due to the fact that the mortality for the Xinjiang breast cancer patients was relatively lower, we further discussed the applicability of the case–cohort design in identifying the prognostic factors of breast cancer patients in Xinjiang, by comparing the comprehensive performance of these models established under the case–cohort and full cohort sampling designs. These results could offer scientific basis for evaluating the prognosis of breast cancer patients in Xinjiang.

## 2 Methods

### 2.1 Case–cohort design

**Figure 1:**
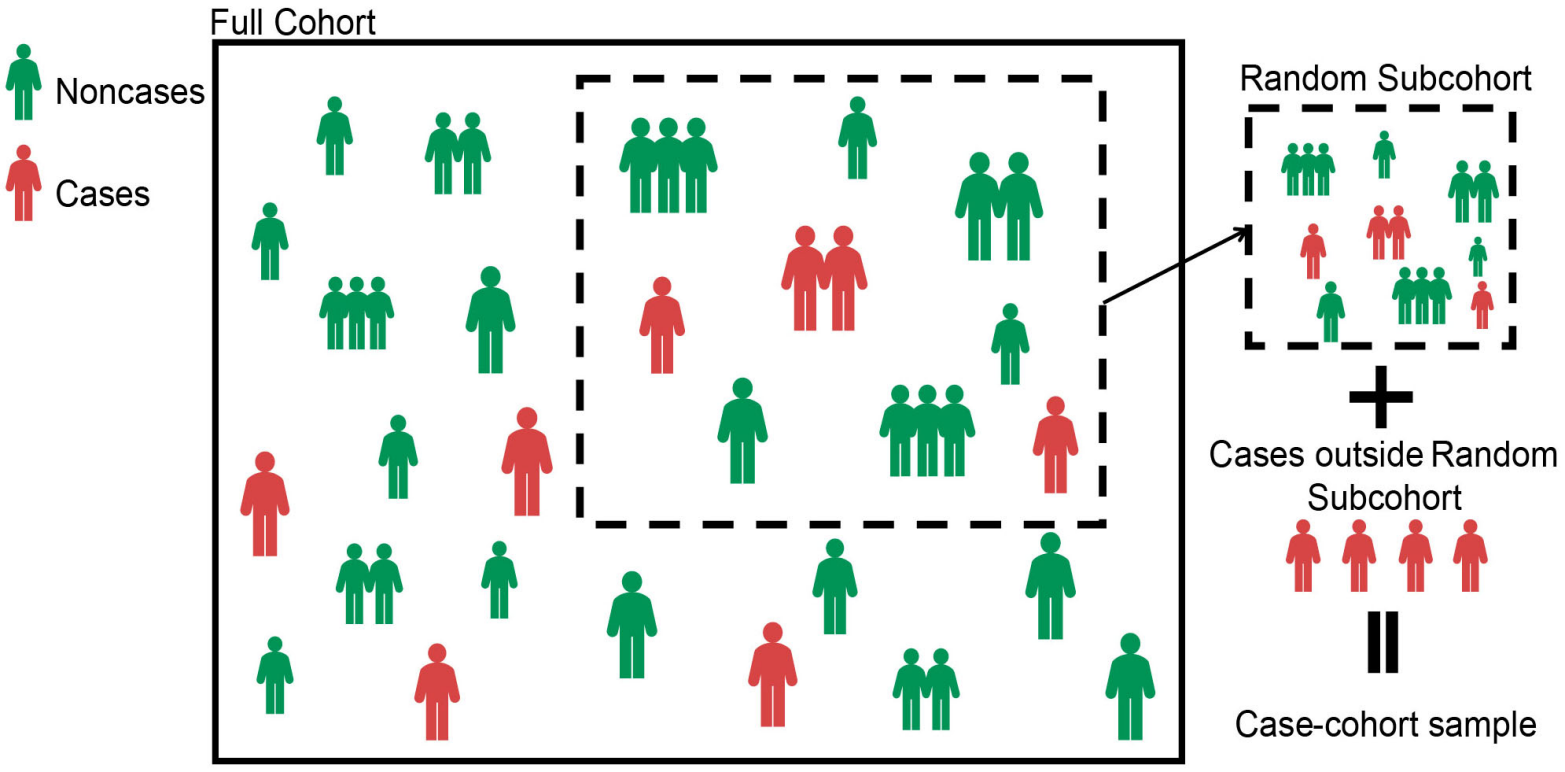
Schematic diagram of the case–cohort design.


In the case–cohort design, the random subcohort (denoted as S ) was selected by simple random sampling from the full cohort. We denoted $S_{i}$ and $\delta_{i}$ as indicator variables, respectively, whether the i th patient was included in the random subcohort and whether the i th patient experienced outcome events. That is, if the i th patient was included in the random subcohort, then $S_{i}=1$ , and if the i th patient experienced the outcome event (i.e., case), then $\delta_{i}=1$ . The case–cohort samples included the random subcohort and all cases outside the random subcohort (20) (see **Figure 1**). Denote $CC_{i}$ as an indicator variable, the explicit expression is as follows,

$$CC_{i}=\{\begin{array}{l} 1,\;\;\;\delta_{i}=1\text{ or }S_{i}=1\;,\\ 0,\;\;\;\delta_{i}=0\text{ and}\;\;S_{i}=0\;. \end{array}$$

In this paper, it is assumed that there are N independent individuals in total. For the survival data with censored, the Cox proportional hazards model is used for analysis. Let X be the covariable for the i th individual and β be the partial regression coefficient, then the basic form of the Cox proportional hazards model is,


$$h_{i}\left(t|\text{X}\right)=h_{0}\left(t\right)\exp\left(\text{Xβ}\right),\;i=1,\ldots ,N,$$

where $h_{0}\left(t\right)$ denotes the baseline risk function. Since the case–cohort design is a biased sampling, the cases and non-cases in the case–cohort design are equally weighted. The pseudolikelihood is used to infer the partial regression coefficient β , then an estimator for β may be obtained by maximizing the pseudolikelihood function


$$L_{p}\left(\text{β}\right)=\prod_{i=1}^{N}{\left[\frac{\exp\left\{{\text{X}}_{i}^{T}{\text{β}}_{i}\right\}}{\sum_{j\in ℛ\left(T_{i}\right)}\exp\left\{{\text{X}}_{j}^{T}{\text{β}}_{j}\right\}}\right]}^{\delta_{i}}\;,$$

where $T_{i}$ represents the observed true event time for i th patient, the risk set at time t denoted by $ℛ\left(t\right)=\left\{\;j:T_{j}\geq t,j\in D\left(t\right)\cup S\;\right\}$ , and D(t) is the collection of cases at time t . Then, the maximum pseudolikelihood estimator for β be solved as


$$U_{p}\left(\text{β}\right)=\sum_{i=1}^{N}\delta_{i}\left[{\text{X}}_{i}-\frac{\sum_{j\in ℛ\left(T_{i}\right)}\exp\left\{{\text{X}}_{j}^{T}{\text{β}}_{j}\right\}X_{j}}{\sum_{j\in ℛ\left(T_{i}\right)}\exp\left\{{\text{X}}_{j}^{T}{\text{β}}_{j}\right\}}\right]=0.$$

### 2.2 Simulation study

Let $T_{i}^{*}$ and $C_{i}$ be the time that the interested event occurs or fails and the time that the i th patient was followed up or censored ( i=1,…,N ), respectively. If $T_{i}^{*}\leq C_{i}$ , then the i th patient experienced the outcome event before the end of the observation period. Otherwise, if $T_{i}^{*}>C_{i}$ , then the i th patient is censored. Thus, the observed true event time is defined as $T_{i}=\min\left(T_{i}^{*},C_{i}\right)$ . Whether or not each patient experienced the outcome event is given by the right censored indicative variable $\delta_{i}=I\left(T_{i}^{*}\leq C_{i}\right)$ , where I(·) is an indicator function.

The time that the interested event occurs or fails for an individual is usually described by using exponential, Weibull, lognormal, and Gamma distributions, etc. The censored time usually follows uniform, exponential distribution and so on (23). In this paper, the survival data were simulated based on the total number of the full cohort sample N=5,000 , $T_{i}^{*}\sim \text{Weibull}\left(\alpha ,\;\lambda \right)$ , and $C_{i}\sim \text{uniform}\left(0,\;\theta \right)$ , where the scale parameter λ=4 , the shape parameter α=2 , and θ denotes as censored rate. Given that this paper mainly focuses on categorical variables, Bernoulli distributions with different probabilities of occurrence were chosen to fit covariates during the simulation process. Therefore, assuming that there are three covariates for each individual, namely, $X_{1}$ , $X_{2},$ and $X_{3}$ , generated from Bernoulli distributions with success rates of 0.1, 0.5, and 0.9, respectively, i.e., $X_{1}\sim B\left(0.1\right)$ , $X_{2}\sim B\left(0.5\right)$ , and $X_{3}\sim B\left(0.9\right)$ (**Table 1**). Then, the Cox proportional hazards model is considered as follows:

**Table 1:** Different sample characteristics of simulated data.

Number	*θ*	Covariates distribution	*β*
1	50%	*X*_1_ ~ *B* (0.1), *X*_2_ ~ *B* (0.5), *X*_3_ ~ *B* (0.9)	(1.5, 1.5, 1.5)^T^
2			(-1.5, -1.5, -1.5)^T^
3	80%	*X*_1_ ~ *B* (0.1), *X*_2_ ~ *B* (0.5), *X*_3_ ~ *B* (0.9)	(1.5, 1.5, 1.5)^T^
4			(-1.5, -1.5, -1.5)^T^
5	90%	*X*_1_ ~ *B* (0.1), *X*_2_ ~ *B* (0.5), *X*_3_ ~ *B* (0.9)	(1.5, 1.5, 1.5)^T^
6			(-1.5, -1.5, -1.5)^T^

θ denotes as censored rate. X_1_, X_2_, and X_3_ indicate covariates. ***β*** denotes the corresponding estimated parameters.


$$h_{i}\left(t\right)=h_{0}\left(t\right)\exp\left(X_{1}\beta_{1}+X_{2}\beta_{2}+X_{3}\beta_{3}\right).$$

The simulated data with six different sample characteristics was simulated based on different censored rates and regression coefficients (**Table 1**).

In the following, we compared the parameter estimations of these sampling designs:

FC: parameter estimations based on full cohort ( ${\hat{\beta }}_{\text{FC}}$ ).

CCI: parameter estimations based on a case–cohort design with one-third proportion sample ( ${\hat{\beta }}_{\text{CCI}}$ ).

CCII: parameter estimations based on a case–cohort design with one-sixth proportion sample ( ${\hat{\beta }}_{\text{CCII}}$ ).

RS: parameter estimations based on random subcohort with one-third proportion sample ( ${\hat{\beta }}_{\text{RS}}$ ).

The simulated data were sampled 1,000 times for parameter estimations. The mean, standard error of the mean (SE.mean), standard deviation (SD), coefficient of variation (CV), range, and bias of these parameters were compared to assess the performance of different sampling designs.

### 2.3 Analysis of breast cancer data

The breast cancer patients collected in this paper was sourced from the Affiliated Cancer Hospital of Xinjiang Medical University. Based on full cohort and case–cohort sampling designs, the survival data of these patients were analyzed to identify the independent prognostic factors of breast cancer patients in Xinjiang, by using Kaplan–Meier analysis, Cox proportional hazards model, and stepwise regression. Meanwhile, the parameter estimations of those models were compared to evaluate the comprehensive performance of these models based on case–cohort and full cohort sampling designs and then assess the effectiveness and clinical value of the case–cohort design.

Potential influencing factors such as survival status (life or death), survival time, basic demographic, and clinicopathological of patients were gathered. The patients’ histological grades of tumors are divided into low, medium, and high. According to immunohistochemical technique, there are luminal A, luminal B, HER2 overexpression, and triple-negative breast cancer. The TNM staging system is divided into T stage (primary tumor), N stage (regional lymph nodes), and M stage (distant organ metastases). T stage was divided into T1 (tumor size, ≤2 cm), T2 (tumor size, 2–5 cm), T3 (tumor size, >5 cm), and T4 (tumors of any size with direct extension to the chest wall and/or to the skin, that is ulceration or skin nodules, macroscopic nodules); N stage included N0 (no regional lymph node metastases), N1 (micrometastases, or metastases in one to three axillary lymph nodes), N2 (metastases in four to nine axillary lymph nodes), and N3 (metastases in 10 or more axillary lymph nodes); and M stage was split into M0 (no clinical or radiographic evidence of distant metastases) and M1 (distant metastases) (24). The types of surgery that patients underwent included no surgery, radical surgery, and breast-conserving surgery. In addition, the age [classified into three categories: younger group (≤45 years), middle-aged group (46–69 years), and the elderly group (≥70 years)] and postoperative chemotherapy of patient were also included.

The inclusion criteria for patients were 1) the age of patient was above 18, 2) tumor of primary site was only identified as breast cancer, and 3) the information of clinicopathological and follow-up were complete. Patients were excluded if 1) medical documents were unsigned, such as informed consent and patient instructions, at the time of admission, and 2) the information about the molecular subtypes, clinical stage, types of surgery, etc., were partial. A total of 8,226 breast cancer patients were followed up in this paper, and the end of the follow-up period was 31 December 2021. Among them, 7,948 patients were effectively followed up, with a follow-up rate of 96.62%. According to the inclusion and exclusion criteria, a total of 3,641 patients were ultimately included, of which 326 patients died (i.e., the censored rate more than 90%).

In this paper, all statistical analysis and visualization were conducted using R 4.1.3 software. A *p*<0.05 based on a two-tailed test was considered statistically significant.

### 2.4 Model evaluation

#### 2.4.1 Likelihood ratio test

The likelihood ratio test was used to evaluate Cox regression models in general and reflect the fitting effect of the models (25), based on the following formula,


$$\chi_{v}^{2}=-2LogL_{i}-\left(-2LogL_{j}\right),$$

where $\chi_{v}^{2}\sim \;\chi^{2}\left(v\right)$ , $-2LogL_{i}$ represents the log-likelihood function value of a regression model with *i* parameters. The smaller the value of $\chi_{v}^{2}$ , the better the fitting effect of the model.

#### 2.4.2 Akaike Information Criterion

Akaike Information Criterion (AIC) (26) is applicable to select the most effective model from various models and evaluate the validity of the modeling results. The general form of this is as follows


AIC =−2ln(L) + 2K ,

where L and K is the maximum likelihood function and the number of independent parameters, respectively. The smaller the AIC value is, which indicates a minimum discrepancy between the probability and the true distribution, the better the model is.

#### 2.4.3 Discrimination

The accuracy of the model predictions is evaluated on the basis of the discrimination. A model showed good discrimination if this model can distinguish whether the patient has reached the endpoint. The area under the receiver operating characteristic (ROC) curve (AUC), which has a value of 0.5–1.0 and the discrimination is better with the higher value of AUC, was used to assess the discrimination of models (27).

## 3 Results

### 3.1 Results of simulation

In the simulation data of six different sample characteristics, the parameters $\beta_{1}$ , $\beta_{2}$ , and $\beta_{3}$ of these models constructed in FC, RS, CCI, and CCII sampling designs were estimated, where full cohort $N=N_{\text{FC}}=5,000$ and subcohort $\;N_{\text{RS}}=1,666$ (see **Table 2**, **Supplementary Tables S1**, **S2**, respectively).

**Table 2:** The simulation results of *β*_1_ under different censored rate and sampling design.

θ	(β_1_, β_2_, β_3_)^T^	Sampling design	β_1_					
			Mean	SE.mean	SD	CV	Range	Bias
50%	(1.5, 1.5, 1.5)^T^	**FC**	1.4998	0.0019	0.0594	0.0396	0.4566	0.0002
		**RS**	1.5064	0.0032	0.1006	0.0668	0.7138	−0.0064
		**CCI**	1.5043	0.0039	0.1218	0.0809	0.8582	−0.0043
		**CCII**	1.5198	0.0053	0.1689	0.1112	1.0474	−0.0198
	(−1.5, −1.5, −1.5)^T^	**FC**	−1.5037	0.0029	0.0933	−0.0620	0.5794	0.0037
		**RS**	−1.5011	0.0050	0.1590	−0.1060	1.1000	0.0011
		**CCI**	−1.4970	0.0048	0.1533	−0.1024	0.9272	−0.0030
		**CCII**	−1.4922	0.0066	0.2074	−0.1390	1.3636	−0.0078
80%	(1.5, 1.5, 1.5)^T^	**FC**	1.4989	0.0025	0.0785	0.0524	0.6556	0.0011
		**RS**	1.5049	0.0043	0.1363	0.0906	1.0314	−0.0049
		**CCI**	1.5062	0.0045	0.1420	0.0943	1.0461	−0.0062
		**CCII**	1.5185	0.0062	0.1963	0.1293	1.1437	−0.0185
	(−1.5, −1.5, −1.5)^T^	**FC**	−1.5094	0.0058	0.1847	−0.1223	1.3911	0.0094
		**RS**	−1.5247	0.0105	0.3306	−0.2168	2.4861	0.0247
		**CCI**	−1.5041	0.0073	0.2311	−0.1537	1.7562	0.0041
		**CCII**	−1.4966	0.0087	0.2749	−0.1837	1.6536	−0.0034
90%	(1.5, 1.5, 1.5)^T^	**FC**	1.4977	0.0032	0.1002	0.0669	0.7329	0.0023
		**RS**	1.5003	0.0058	0.1847	0.1231	1.2513	−0.0003
		**CCI**	1.5030	0.0050	0.1589	0.1057	1.2128	−0.0030
		**CCII**	1.5134	0.0068	0.2137	0.1412	1.2151	−0.0134
	(−1.5, −1.5, −1.5)^T^	**FC**	−1.5420	0.0091	0.2888	−0.1873	1.8267	0.0420
		**RS**	−1.7807	0.0570	1.8040	−1.0131	17.9073	0.2807
		**CCI**	−1.5379	0.0102	0.3225	−0.2097	1.9279	0.0379
		**CCII**	−1.5257	0.0111	0.3510	−0.2301	2.5577	0.0257

θ denotes as censored rate. β_1_, β_2_, and β_3_ indicate the estimated parameters.

SE, mean standard error of the mean; SD, standard deviation; CV, coefficient of variation; FC, full cohort; RS, random subcohort; CCI, case–cohort design with one-third proportion sample; CCII, case–cohort design with one-sixth proportion sample.

The estimated results of $\beta_{1}$ , $\beta_{2},$ and $\beta_{3}$ showed that its mean values were relatively close under different parameter settings of four sampling designs. Its SE.mean, SD, CV, range, and bias were small, which demonstrated that Cox proportional hazards model presents the better ability in the analysis of the simulated data. Moreover, the findings showed that the fitting results of parameters in the RS and CCI sampling designs approached to the same with a large bias in the results of CCII sampling design when θ=50% . For instance, in the scenario of θ=50% and $\beta_{1}=\beta_{2}=\beta_{3}=-1.5$ (**Supplementary Table S2**), the bias value of $\beta_{3}$ in CCII is approximately 0.02, and its SD, CV, and range are also larger than those of other sampling designs.

**Figure 2:**
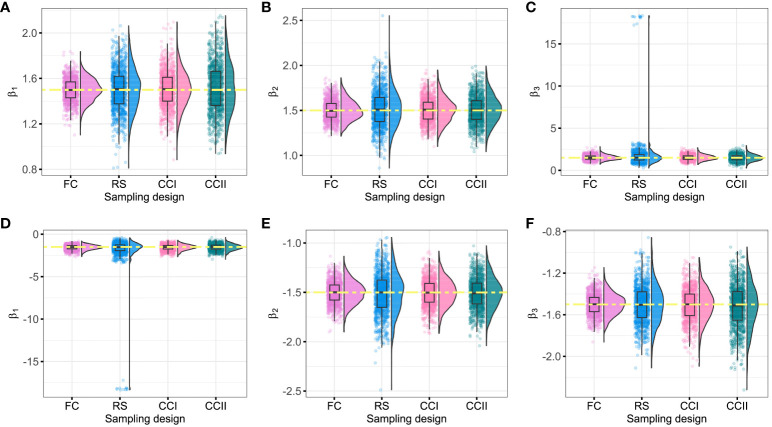
Fitting values of *β*_1_, *β*_2_, and *β*_3_ under different sampling designs (*θ* = 90%). The yellow dashed line represents the initial value of the regression coefficients. **(A–C)** The fitting values of *β*_1_, *β*_2_, and *β*_3_ when the initial regression coefficients are 1.5; **(D–F)** the fitting values of *β*_1_, *β*_2_, and *β*_3_ when the initial regression coefficients are −1.5. FC, full cohort; RS, random subcohort; CCI, case–cohort design with one-third proportion sample; CCII, case–cohort design with one-sixth proportion sample.

On the other hand, it was found that when the censored rate increases, the efficiency of simple random sampling design decreases, the range, SD, and CV of parameter estimations under this sampling design become larger, and then the possibility of outlier is increased. In actual application, there may be a large bias in the results of simple random sampling design without repeated sampling. For instance, under the RS sampling design, when θ=90% , $\beta_{1}=\beta_{2}=\beta_{3}=-1.5$ (**Figure 2C**) or 1.5 (**Figure 2D**), respectively, there are many outliers with the ranges of approximately 18 in the fitted values of $\beta_{3}$ and $\beta_{1}$ , which greatly exceeds the ranges of the estimated values under other sampling designs.

Moreover, when the censored rate is high (i.e., θ=80% or θ=90% ), CCI and CCII sampling designs have good stability, with smaller dispersion degree and variation index of the parameters, especially CCI. CCI sampling design improves the estimation efficiency because only partial samples (approximately 40%) of the full cohort samples were used by this sampling design to reach the fitting result of FC sampling design, as shown in **Table 2**, **Figure 2**, and **Supplementary Figures S1**, **S2**. Therefore, when the sample censored rate was 90%, the sample error of the case–cohort design is smaller than that of simple random sampling.

### 3.2 Results of breast cancer data

**Figure 3:**
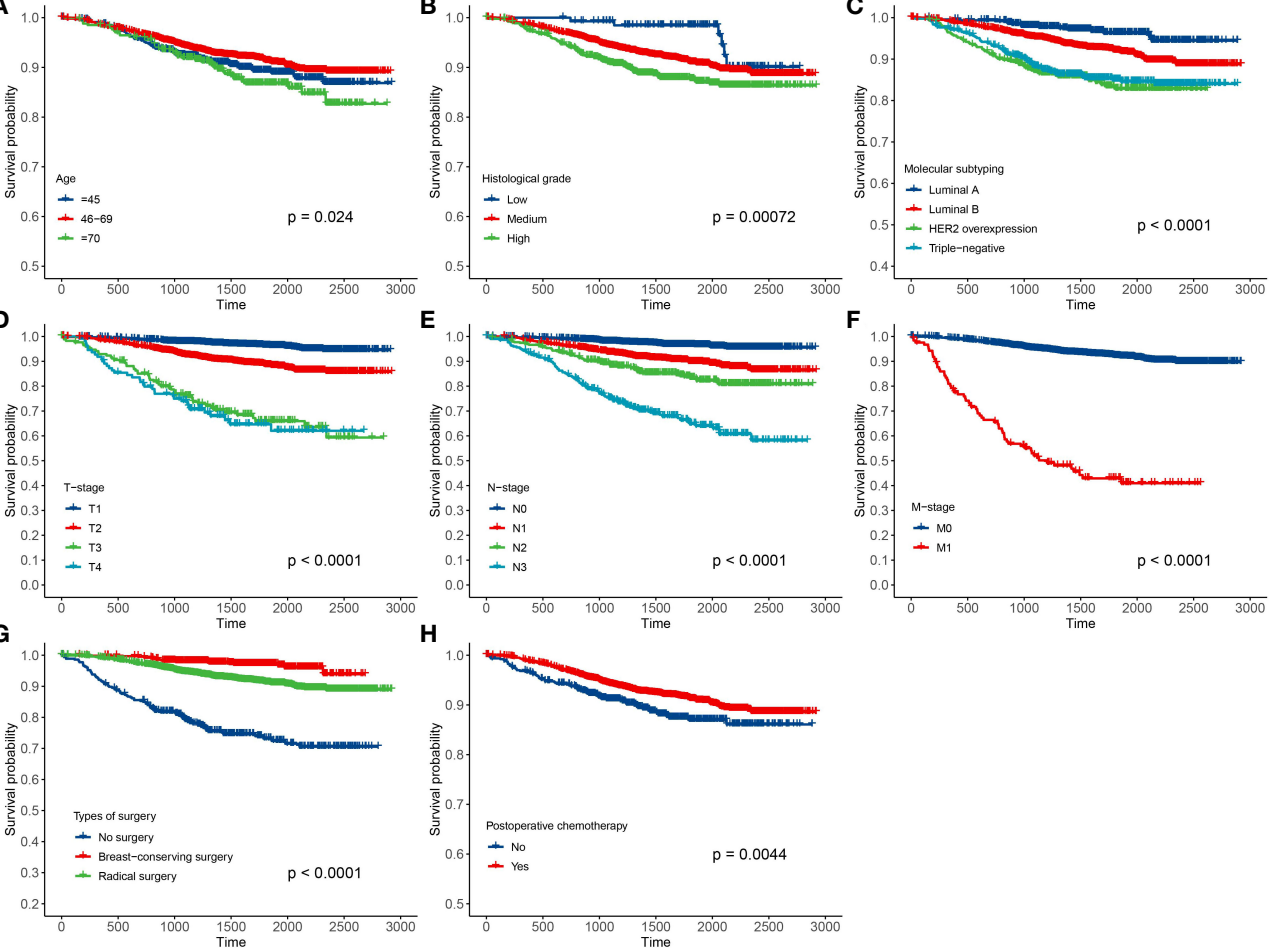
Results of Kaplan–Meier analysis for the clinical data of breast cancer patients based on the full cohort sampling designs. **(A)** Age; **(B)** histological grade; **(C)** molecular subtyping; **(D)** T stage; **(E)** N stage; **(F)** M stage; **(G)** types of surgery; and **(H)** postoperative chemotherapy.

**Figure 4:**
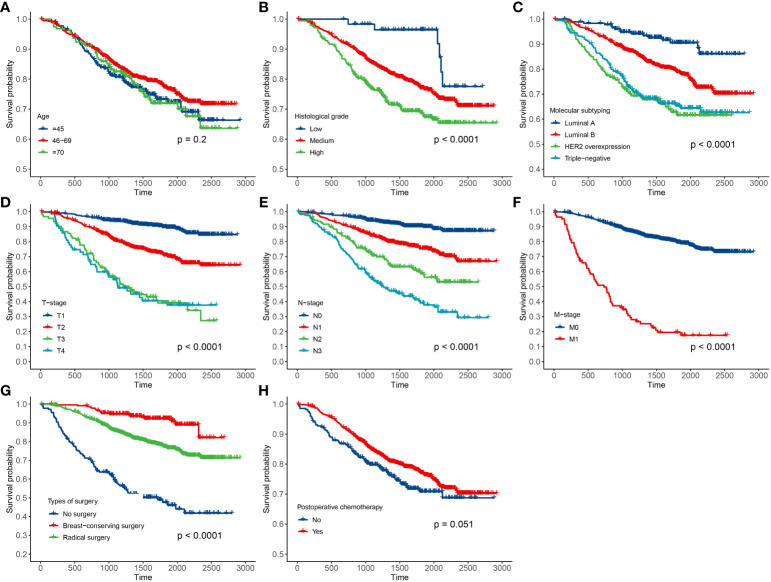
Results of Kaplan–Meier analysis for the clinical data of breast cancer patients based on the case–cohort sampling designs. **(A)** Age; **(B)** histological grade; **(C)** molecular subtyping; **(D)** T stage; **(E)** N stage; **(F)** M stage; **(G)** types of surgery; and **(H)** postoperative chemotherapy.

In this paper, there were 3,641 breast cancer patients in Xinjiang with a censored rate of more than 90% as full cohort samples, of which only 326 patients experienced the outcome event (i.e., death). Hence, based on the results of the simulation in *Section 3.1*, the case–cohort design with a one-third sample proportion was selected to analyze these data. First, one-third of the patients were randomly selected as a random subcohort (1,214 patients) combining with all cases outside the subcohort, and then, a case–cohort sample with 1,418 patients was formed. The basic information about clinicopathological characteristics of patients is shown in **Table 3**. Furthermore, Kaplan–Meier analysis was performed to analyze the clinical data of patients based on the full cohort and case–cohort sampling designs, as shown in **Figures 3** and **4**, respectively. Then, the statistically significant factors (*p*< 0.05) in Kaplan–Meier analysis and factors with clinical practice value were added to the Cox regression model, and the significant prognostic factors were selected by bidirectional stepwise regression.

**Table 3:** Basic information about clinicopathological characteristics of breast cancer patients in Xinjiang.

	Full cohort	Case–cohort
	(N=3641)	(N=1418)
Survival time		
$\bar{x}\pm sd$	1,630 ± 578	1,520 ± 616
Survival state		
Survival	3,315(91.0%)	1,092(77.0%)
Death	326(9.0%)	326(23.0%)
Age		
≤45	691(19.0%)	273(19.3%)
46-69	2,609(71.7%)	988(69.7%)
≥70	341(9.4%)	157(11.1%)
Histological grade		
Low	138 (3.8%)	60 (4.2%)
Medium	2,606 (71.6%)	1,007 (71.0%)
High	897 (24.6%)	351 (24.8%)
Molecular subtyping		
Luminal A	473 (13.0%)	182 (12.8%)
Luminal B	2,218 (60.9%)	824 (58.1%)
HER2 overexpression	397 (10.9%)	175 (12.3%)
Triple-negative	553 (15.2%)	237 (16.7%)
T-stage		
T1	1,740 (47.8%)	632 (44.6%)
T2	1,620 (44.5%)	633 (44.6%)
T3	171 (4.7%)	90 (6.3%)
T4	110 (3.0%)	63 (4.4%)
N-stage		
N0	1,814 (49.8%)	636 (44.9%)
N1	1,140 (31.3%)	463 (32.7%)
N2	389 (10.7%)	156 (11.0%)
N3	298 (8.2%)	163 (11.5%)
M-stage		
M0	3,518 (96.6%)	1,335 (94.1%)
M1	123 (3.4%)	83 (5.9%)
Types of surgery		
No surgery	357 (9.8%)	179 (12.6%)
Breast-conserving surgery	630 (17.3%)	230 (16.2%)
Radical surgery	2,654 (72.9%)	1,009 (71.2%)
Postoperative chemotherapy		
No	635 (17.4%)	275 (19.4%)
Yes	3,006 (82.6%)	1,143 (80.6%)

$\bar{x}\pm sd$ denotes mean ± standard deviation.

**Figure 5:**
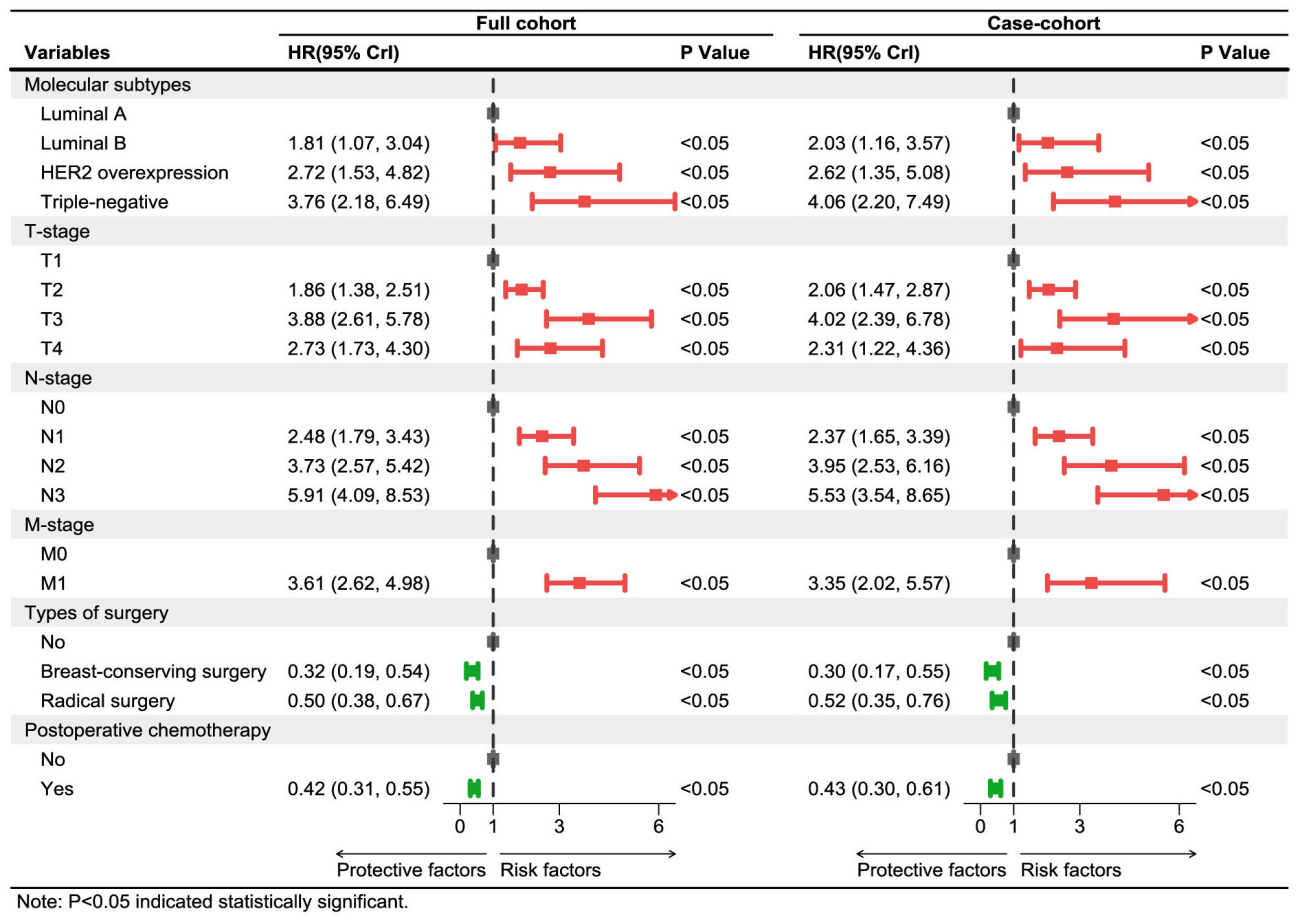
Multivariate Cox regression models of breast cancer patients in Xinjiang with full cohort and case–cohort sampling designs. The red lines and squares reflect the HR and 95%CrI for risk factors, while green reflects the HR and 95%CrI for protective factors. HR, hazard ratio; CrI, credibility interval.


The fitting results of Cox regression model showed that the parameter estimations under the two sampling designs were very close (see **Figure 5**). It was finally determined that molecular subtypes, T stage, N stage, and M stage were the risk factors for prognosis of Xinjiang breast cancer patients (*p*<0.05 and HR>1). In detail, patients with clinicopathological features of triple-negative breast cancer, T3, N3, and M1 substages had the highest risk of death. Simultaneously, types of surgery and postoperative chemotherapy were protective factors for independent prognosis (*p*<0.05 and HR<1). Patients who underwent breast-conserving surgery, radical surgery, and postoperative chemotherapy had a lower risk of death than others who did not have surgery. Thus, a model that can effectively predict prognosis of patients has been established as follows:

$$h\left(t|X\right)=h_{0}\left(t\right)\exp\left(\beta_{1}X_{\text{Molecular subtypes}}+\beta_{2}X_{\text{T-stage}}+\beta_{3}X_{\text{N-stage}}+\beta_{4}X_{\text{M-stage}}+\beta_{5}X_{\text{Types of surgery }}+\beta_{6}X_{\text{Postoperative chemotherapy}}\right).$$

Finally, the performances of these models established on the basis of the case–cohort (CCI) and full cohort (FC) sampling designs were comprehensively evaluated, as shown in **Table 4**. Both Cox proportional hazards models established under the two sampling designs passed the likelihood ratio test (*p*<0.05), where $\chi_{v\;\text{CCI}}^{2}=490.05<\;\chi_{v\;\text{FC}}^{2}=518.80$ . In addition, the AIC value (3,999.96) obtained by the CCI was also smaller compared with the FC, which indicated that the fitting effect of the case–cohort sampling design was better.

**Table 4:** Evaluation indexes of Cox regression models under different sampling designs.

Sampling designs	Likelihood ratio test		AIC	AUC (95%CrI)
	$\chi_{v}^{2}$	*p*-value		
Full cohort (**FC**)	518.80	<0.05^a^	4690.78	0.805 (0.779, 0.832)
Case-cohort (**CCI**)	490.05	<0.05^a^	3999.96	0.807 (0.780, 0.835)

AIC, Akaike Information Criterion; AUC, the area under the receiver operating characteristic curve. CrI, credible interval.

a p<0.05 indicates statistically significant. $\chi_{v}^{2}$ denotes the value of chi-square test.

**Figure 6:**
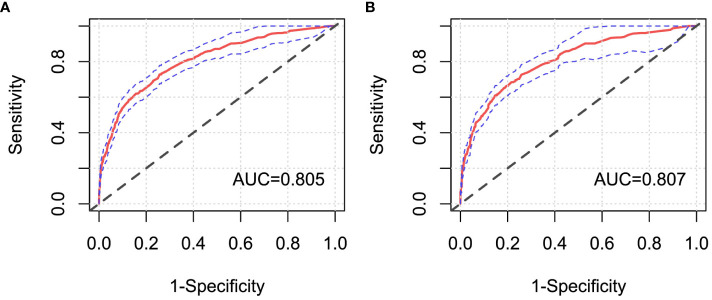
ROC curves of Cox regression models under different sampling designs. **(A)** Full cohort design; **(B)** case–cohort design. AUC, the area under the receiver operating characteristic curve.

Moreover, ROC curves of Cox regression models under FC and CCI sampling designs were separately drawn to compare the discrimination of these models (**Figure 6**). It was shown that both AUC values were >0.8, and they were very close, which confirmed a good discrimination for the prognostic model constructed in this paper and also further verified that the case–cohort sampling design reached a better fitting effect only using approximately 38.9% of the full cohort samples.

## 4 Discussion

Case–cohort design is suitable for cancer research with large cohort and low incidence, which could improve efficiency and reduce the cost of collecting redundant non-case data (18). One of the highlights of this paper is that the effectiveness of the case–cohort design was verified based on the Cox proportional risk model, and the different censored rates (50%, 80%, and 90%) and different sampling ratios (1/3, 1/6) were conducted in the simulation study. By simulating the survival data with different sample characteristics, this study estimated the coefficients of Cox regression models in FC, CCI, CCII, and RS sampling designs to assess the performance of the models and sampling designs, respectively. Our findings showed that the case–cohort design could improve the estimation efficiency, especially the higher censored rate. Since the morbidity of breast cancer has been an increasing tendency year by year in Xinjiang (10), and the mortality for the followed up Xinjiang breast cancer patients was relatively lower, using the case–cohort design could reduce the bias caused by random sampling, more effectively identify prognostic factors, and further promote the improvement of clinical prognostic methods. Therefore, based on the case–cohort design, this study analyzed the actual clinical data of breast cancer patients in Xinjiang to identify independent prognostic factors (molecular subtypes, T stage, N stage, M stage, types of surgery, and postoperative chemotherapy). Another innovation of this paper is that the performance of the model established under the full cohort and case–cohort in the actual data were comprehensively evaluated in breast cancer patients in Xinjiang by likelihood ratio test, AIC criterion, and discrimination. This further confirmed that the prognosis model constructed under the case–cohort sampling design had better fitting effect than that based on the full cohort sampling design, and the case–cohort sampling design showed certain applicability in the actual data.

The results of simulations in this paper displayed that the estimated mean values of regression coefficients were close to the given initial values in the survival data with different scenarios, indicating that Cox proportional hazards model could achieve the better fitting effect. In addition, when the censored rate was lower, the fitting results of the regression coefficients under the RS and CCI sampling designs were nearly the same, while there was a lager bias of the parameter estimations under CCII sampling designs. It demonstrated that not only the suitable sampling designs should be selected but also the sampling proportion should not be too small in the analysis; otherwise, it would also reduce the statistical efficiency. On the other hand, when the censored rate gradually increased, the parameter estimations under the single simple random sampling design would be more likely to generate outliers, which could result in the gradual decrease in efficiency under this sampling design. However, in actual applications, it is often difficult to conduct multiply repeated sampling, which may lead to a significant deviation in the obtained results. Meanwhile, our findings revealed that when the censored rate was higher, the CCI and CCII sampling designs had superior stability (i.e., there are fewer outliers and smaller deviations), especially CCI sampling design. Both estimated mean values under CCI and CCII sampling designs had smaller dispersion degree and variation index, and the CCI design results that only used 38.9% samples of the full cohort samples were close to FC design results. Moreover, using different types of covariates may have a certain influence on the simulation results, but this impact is relatively small, as demonstrated in the paper by Yang et al. (20), where there were slight differences between simulation results of the normal and uniform distribution. To sum up, the simulation results of this paper confirmed that the case–cohort design is a cost-effective sampling design compared with simple random sampling design, which could improve the efficiency of estimation. In particular, the case–cohort design was more effective and stable when the interested events had a relatively lower incidence, which was consistent with the results in these references (18, 20, 28).

In this paper, the breast cancer patients followed up were registered in the Affiliated Cancer Hospital of Xinjiang Medical University, which could be regarded as a random sample from the overall population, with more than 90% censored rate. Thus, a one-third proportion of case–cohort sampling design was used to analyze these data, and the same Cox regression model was also simultaneously implemented in the full cohort sampling design to compare the difference between the two designs’ results. The results showed that the prognosis of patients with triple-negative breast cancer was the worst, which may be the cause of the tumor cells of those patients being more aggressive and more prone to recurrence and metastasis (29). Luminal breast cancer patients had better prognosis and higher survival rate than other non-luminal patients. Moreover, T, N, and M stages were independent risk prognostic factors of breast cancer patients. Patients with advanced T stage had larger tumors, more tumor cells, and the longer time for the tumor formation, so these patients would be more likely to develop into distant metastasis breast cancer ones. The later stage of N stage indicated greater probability, more numbers of lymph node metastases, and higher risk of death, which are typical clinical features of breast cancer progression (30). Because distant metastasis of breast cancer (i.e., M stage) means that the tumors of breast cancer could spread to the lung, liver, brain, and other parts of the body, the occurrence of distant metastasis (i.e., advanced breast cancer) would result in more difficult clinical treatment (31). Therefore, regular breast self-examination and clinical screening for women were recommended to achieve the purpose of early detection, early diagnosis, and early treatment, and then reduce the mortality and improve the prognosis of breast cancer patients. At the same time, it was also shown that the breast-conserving surgery [HR=0.30, 95%CrI: (0.17, 0.55)], radical surgery [HR=0.52, 95%CrI: (0.35, 0.76)], and postoperative chemotherapy [HR=0.43, 95%CrI: (0.30, 0.61)] were protective factors for breast cancer patients in Xinjiang. These surgeries could effectively reduce the size of the tumor, reduce the number of tumors, and control the spread of the disease, thereby greatly improving the survival probability for breast cancer patients. Initially, the radical surgery, as a common treatment, occupied a very important position. But now, breast-conserving surgery is more widely used to treat patients with early disease progression, with the characteristics of shorter operation time and lower incidence of postoperative complications (32). Standard postoperative adjuvant chemotherapy for patients could prevent the recurrence and control the metastasis of cancer to a certain extent, and it could reduce the pain, improve the quality of life, and then extend their life cycle for some patients with advanced stage (33). Finally, the likelihood ratio test, ROC curve, and AIC criteria were used to compare the superiority of model prediction in the full cohort and the case–cohort sampling designs. The comparison findings showed that both models under FC and CCI sampling designs passed the likelihood ratio test (*p*<0.05), and the model constructed under the CCI design had better fitting effect (AIC=3,999.96) and better discrimination [AUC=0.807, 95%CrI: (0.780, 0.835)], which demonstrated that the case–cohort design was suitable to analyze the prognosis of breast cancer patients in Xinjiang.

There are some limitations in this study. On the one hand, we only employed Cox proportional hazards model with Prentice’s weight method to investigate the effectivity and stability of the case–cohort design. However, different weighted estimation methods (such as Barlow and Self-Prentice method) or different statistical models (such as additive risk model) could also be applied to make statistical inference to be more accurate and effective under the case–cohort design when the weights of case–cohort samples are not mutually independent or the actual data do not follow the proportional hazards assumption. On the other hand, only the clinical data of breast cancer patients in Xinjiang were analyzed in this paper, but the applicability of the case–cohort design in the other regions or other cancers deserves to be further explored. Last but not least, the main purpose of our paper is to explore the factors affecting the prognosis of breast cancer patients in Xinjiang, based on the case–cohort design and Cox proportional risk model. Hence, we focused on the influence degree of different factors on the occurrence time of the event. It was needed to consider the impact of covariates on survival time and the chronological order of events; therefore, we only reported HR values in the outcome in this paper. In our future work, we will consider different methods such as logistic regression or propensity score to calculate different statistical indicators (such as OR and RR values) (34–36), in order to find the best reporting indicator for actual data with different sample characteristics.

## 5 Conclusion

In summary, this study demonstrated the effectivity and stability of the case–cohort design through simulating data and confirmed that this design could maintain a better estimation efficiency in cancers with high censored rate. Furthermore, independent prognostic factors of breast cancer patients in Xinjiang were determined under the case–cohort design, and the practical fitting effect and useful application of the case–cohort design were demonstrated by comparing with the results based on full cohort design.

## Data availability statement

The data that support the findings of this study are available from the Affiliated Cancer Hospital of Xinjiang Medical University but restrictions apply to the availability of these data, which were used under license for the current study, and so are not publicly available. Requests to access the datasets should be directed to TiZ, zhaoting0557@163.com.

## Ethics statement

The studies involving human participants were reviewed and approved by Medical Ethics Committee of the Affiliated Cancer Hospital of Xinjiang Medical University (approval number: K-2023001). The participants provided their written informed consent to participate in this study.

## Author contributions

MW: Conceptualization, Software, Writing – original draft, Writing – review & editing. GS: Data curation, Funding acquisition, Writing – review & editing. TaZ: Software, Writing – original draft. CG: Methodology, Writing – original draft. TiZ: Data curation, Resources, Writing – review & editing. QZ: Data curation, Resources, Writing – review & editing. LW: Conceptualization, Methodology, Writing – original draft, Writing – review & editing.
