# Supplementary material for: Assessing of case–cohort design: a case study for breast cancer patients in Xinjiang, China
Source: Front Oncol. 2024 Mar 20;14:1306255. doi: 10.3389/fonc.2024.1306255 (PMC10987809; doi:10.3389/fonc.2024.1306255)
Supplement: Supplementary file 1 [file DataSheet_1.docx]

Supplementary Material

Assessing of case-cohort design: a case study for breast cancer patients in Xinjiang, China

# Supplementary Figures and Tables

## Supplementary Figures


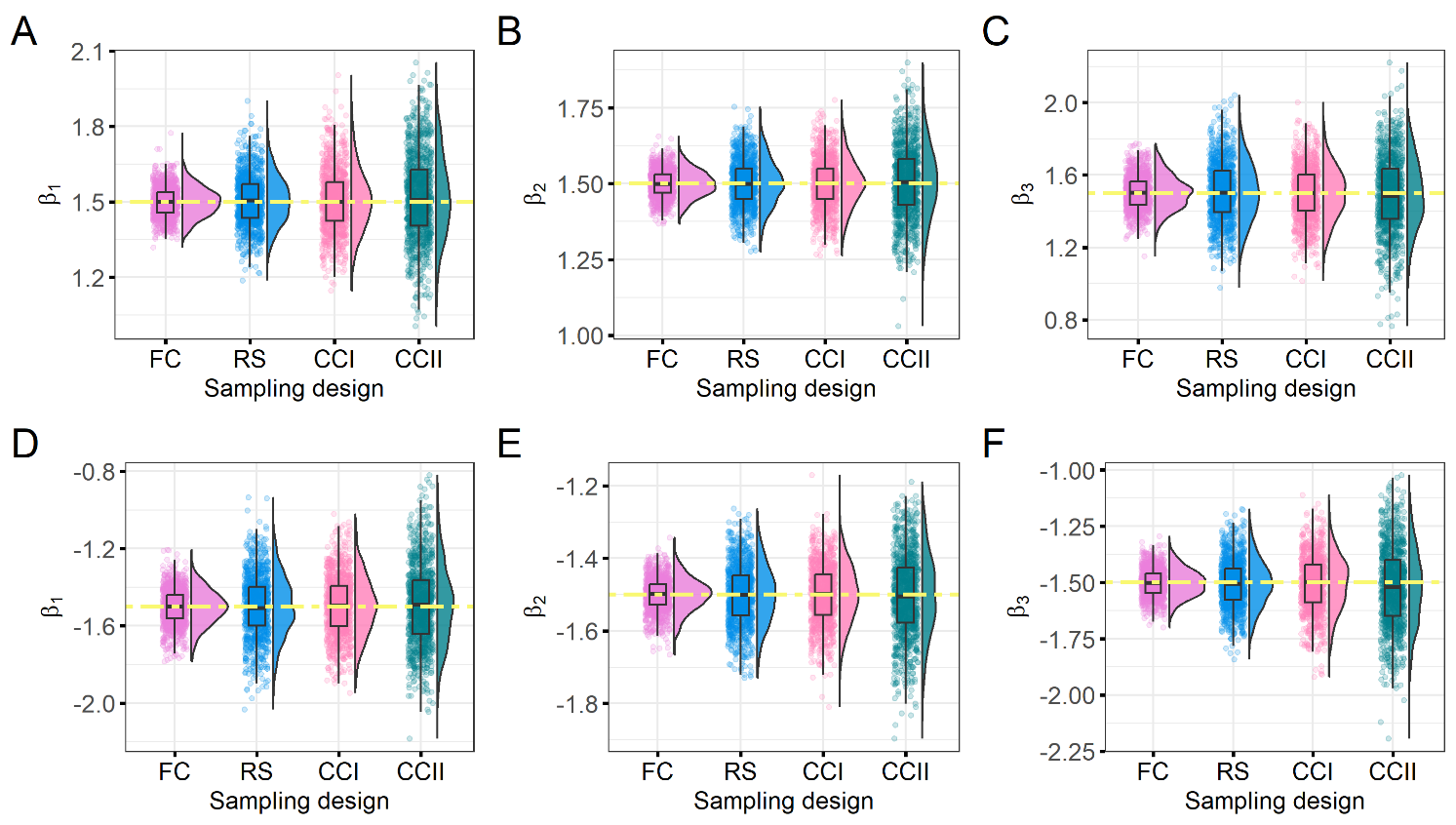


Supplementary Figure 1. Fitting values of $\boldsymbol{\beta}_{\text{1}}$, $\boldsymbol{\beta}_{\text{2}}$ and $\boldsymbol{\beta}_{\text{3}}$ under different sampling designs ($\boldsymbol{\theta=}\text{50\%}$). The yellow dashed line represents the initial value of the regression coefficients. (A-C) The fitting values of $\boldsymbol{\beta}_{\text{1}}$, $\boldsymbol{\beta}_{\text{2}}$ and $\boldsymbol{\beta}_{\text{3}}$ when the initial regression coefficients are 1.5; (D-F) The fitting values of $\boldsymbol{\beta}_{\text{1}}$, $\boldsymbol{\beta}_{\text{2}}$ and $\boldsymbol{\beta}_{\text{3}}$ when the initial regression coefficients are -1.5. FC, full cohort; RS, random subcohort; CCI, case-cohort design with one-third proportion sample; CCII, case-cohort design with one-sixth proportion sample.


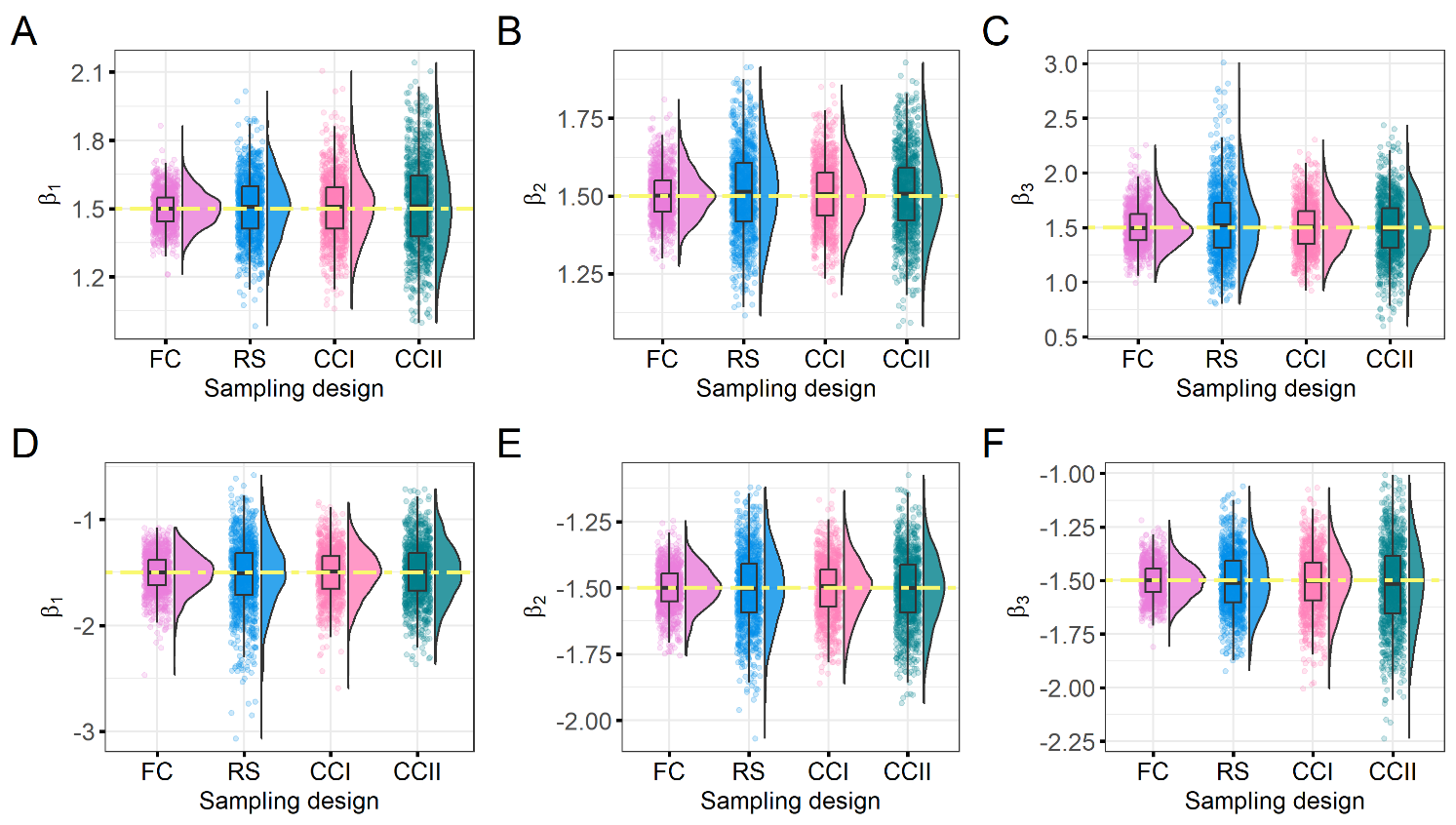


Supplementary Figure 2. Fitting values of $\boldsymbol{\beta}_{\text{1}}$, $\boldsymbol{\beta}_{\text{2}}$ and $\boldsymbol{\beta}_{\text{3}}$ under different sampling designs ($\boldsymbol{\theta=}\text{80\%}$). The yellow dashed line represents the initial value of the regression coefficients. (A-C) The fitting values of $\boldsymbol{\beta}_{\text{1}}$, $\boldsymbol{\beta}_{\text{2}}$ and $\boldsymbol{\beta}_{\text{3}}$ when the initial regression coefficients are 1.5; (D-F) The fitting values of $\boldsymbol{\beta}_{\text{1}}$, $\boldsymbol{\beta}_{\text{2}}$ and $\boldsymbol{\beta}_{\text{3}}$ when the initial regression coefficients are -1.5. FC, full cohort; RS, random subcohort; CCI, case-cohort design with one-third proportion sample; CCII, case-cohort design with one-sixth proportion sample.

## Supplementary Tables

Supplementary Table 1. The simulation results of $\boldsymbol{\beta}_{\text{2}}$ under different censored rate and sampling design.

| $\theta$ | ${(\beta_{\text{1}}, \beta_{\text{2}}, \beta_{\text{3}})}^{\text{T}}$ | Sampling  design | $\beta_{\text{2}}$ | | | | | |
| --- | --- | --- | --- | --- | --- | --- | --- | --- |
|  |  |  | Mean | SE.mean | SD | CV | Range | Bias |
| 50% | (1.5, 1.5, 1.5)^T^ | **FC** | 1.4995 | 0.0014 | 0.0447 | 0.0298 | 0.2889 | 0.0005 |
|  |  | **RS** | 1.5019 | 0.0024 | 0.0757 | 0.0504 | 0.4768 | -0.0019 |
|  |  | **CCI** | 1.5010 | 0.0025 | 0.0804 | 0.0536 | 0.5140 | -0.0010 |
|  |  | **CCII** | 1.5054 | 0.0037 | 0.1165 | 0.0774 | 0.8680 | -0.0054 |
|  | (-1.5, -1.5, -1.5)^T^ | **FC** | -1.5005 | 0.0014 | 0.0457 | -0.0305 | 0.3230 | 0.0005 |
|  |  | **RS** | -1.5013 | 0.0026 | 0.0818 | -0.0545 | 0.4671 | 0.0013 |
|  |  | **CCI** | -1.5004 | 0.0026 | 0.0813 | -0.0542 | 0.6402 | 0.0004 |
|  |  | **CCII** | -1.5025 | 0.0036 | 0.1129 | -0.0751 | 0.7075 | 0.0025 |
| 80% | (1.5, 1.5, 1.5)^T^ | **FC** | 1.5040 | 0.0024 | 0.0768 | 0.0511 | 0.5352 | -0.0040 |
|  |  | **RS** | 1.5122 | 0.0043 | 0.1367 | 0.0904 | 0.7992 | -0.0122 |
|  |  | **CCI** | 1.5066 | 0.0033 | 0.1028 | 0.0682 | 0.6754 | -0.0066 |
|  |  | **CCII** | 1.5095 | 0.0041 | 0.1309 | 0.0867 | 0.8466 | -0.0095 |
|  | (-1.5, -1.5, -1.5)^T^ | **FC** | -1.5011 | 0.0025 | 0.0794 | -0.0529 | 0.5094 | 0.0011 |
|  |  | **RS** | -1.5045 | 0.0044 | 0.1401 | -0.0931 | 0.9481 | 0.0045 |
|  |  | **CCI** | -1.5019 | 0.0034 | 0.1082 | -0.0720 | 0.7260 | 0.0019 |
|  |  | **CCII** | -1.5065 | 0.0042 | 0.1339 | -0.0889 | 0.8610 | 0.0065 |
| 90% | (1.5, 1.5, 1.5)^T^ | **FC** | 1.5030 | 0.0035 | 0.1113 | 0.0741 | 0.6478 | -0.0030 |
|  |  | **RS** | 1.5151 | 0.0062 | 0.1976 | 0.1304 | 1.6923 | -0.0151 |
|  |  | **CCI** | 1.5049 | 0.0042 | 0.1335 | 0.0887 | 0.8503 | -0.0049 |
|  |  | **CCII** | 1.5085 | 0.0049 | 0.1546 | 0.1025 | 0.9495 | -0.0085 |
|  | (-1.5, -1.5, -1.5)^T^ | **FC** | -1.5025 | 0.0036 | 0.1133 | -0.0754 | 0.7670 | 0.0025 |
|  |  | **RS** | -1.5123 | 0.0066 | 0.2084 | -0.1378 | 1.5436 | 0.0123 |
|  |  | **CCI** | -1.5034 | 0.0043 | 0.1353 | -0.0900 | 0.8326 | 0.0034 |
|  |  | **CCII** | -1.5085 | 0.0050 | 0.1569 | -0.1040 | 1.0122 | 0.0085 |

$\theta$ denotes as censored rate. $\beta_{\text{1}}, \beta_{\text{2}}, \beta_{\text{3}}$ indicate the estimated parameters.

Abbreviations: SE.mean, standard error of the mean, SD standard deviation; CV, coefficient of variation; FC, full cohort; RS, random subcohort; CCI, case-cohort design with one-third proportion sample; CCII, case-cohort design with one-sixth proportion sample.

Supplementary Table 2. The simulation results of $\boldsymbol{\beta}_{\text{3}}$ under different censored rate and sampling design.

| $\theta$ | ${(\beta_{\text{1}}, \beta_{\text{2}}, \beta_{\text{3}})}^{\text{T}}$ | Sampling  design | $\beta_{\text{3}}$ | | | | | |
| --- | --- | --- | --- | --- | --- | --- | --- | --- |
|  |  |  | Mean | SE.mean | SD | CV | Range | Bias |
| 50% | (1.5, 1.5, 1.5)^T^ | **FC** | 1.5029 | 0.0030 | 0.0940 | 0.0626 | 0.6219 | -0.0029 |
|  |  | **RS** | 1.5088 | 0.0052 | 0.1650 | 0.1094 | 1.0642 | -0.0088 |
|  |  | **CCI** | 1.4997 | 0.0046 | 0.1467 | 0.0978 | 0.9874 | 0.0003 |
|  |  | **CCII** | 1.4910 | 0.0065 | 0.2059 | 0.1381 | 1.4550 | 0.0090 |
|  | (-1.5, -1.5, -1.5)^T^ | **FC** | -1.5018 | 0.0020 | 0.0621 | -0.0414 | 0.4034 | 0.0018 |
|  |  | **RS** | -1.5049 | 0.0034 | 0.1071 | -0.0712 | 0.6686 | 0.0049 |
|  |  | **CCI** | -1.5055 | 0.0039 | 0.1233 | -0.0819 | 0.8076 | 0.0055 |
|  |  | **CCII** | -1.5202 | 0.0056 | 0.1768 | -0.1163 | 1.1705 | 0.0202 |
| 80% | (1.5, 1.5, 1.5)^T^ | **FC** | 1.5103 | 0.0058 | 0.1830 | 0.1212 | 1.2609 | -0.0103 |
|  |  | **RS** | 1.5409 | 0.0102 | 0.3233 | 0.2098 | 2.2057 | -0.0409 |
|  |  | **CCI** | 1.5104 | 0.0069 | 0.2182 | 0.1445 | 1.3804 | -0.0104 |
|  |  | **CCII** | 1.4879 | 0.0087 | 0.2741 | 0.1842 | 1.8343 | 0.0121 |
|  | (-1.5, -1.5, -1.5)^T^ | **FC** | -1.5004 | 0.0026 | 0.0813 | -0.0542 | 0.5922 | 0.0004 |
|  |  | **RS** | -1.5051 | 0.0044 | 0.1388 | -0.0923 | 0.8617 | 0.0051 |
|  |  | **CCI** | -1.5065 | 0.0044 | 0.1398 | -0.0928 | 0.9372 | 0.0065 |
|  |  | **CCII** | -1.5233 | 0.0062 | 0.1964 | -0.1289 | 1.2298 | 0.0233 |
| 90% | (1.5, 1.5, 1.5)^T^ | **FC** | 1.5247 | 0.0089 | 0.2807 | 0.1841 | 2.0832 | -0.0247 |
|  |  | **RS** | 1.7743 | 0.0590 | 1.8672 | 1.0524 | 18.0695 | -0.2743 |
|  |  | **CCI** | 1.5238 | 0.0097 | 0.3057 | 0.2006 | 1.9928 | -0.0238 |
|  |  | **CCII** | 1.4994 | 0.0113 | 0.3559 | 0.2374 | 2.6664 | 0.0006 |
|  | (-1.5, -1.5, -1.5)^T^ | **FC** | -1.4991 | 0.0032 | 0.1021 | -0.0681 | 0.7150 | -0.0009 |
|  |  | **RS** | -1.5027 | 0.0057 | 0.1791 | -0.1192 | 1.2533 | 0.0027 |
|  |  | **CCI** | -1.5035 | 0.0049 | 0.1543 | -0.1026 | 1.0451 | 0.0035 |
|  |  | **CCII** | -1.5182 | 0.0066 | 0.2074 | -0.1366 | 1.3722 | 0.0182 |

$\theta$ denotes as censored rate. $\beta_{\text{1}}, \beta_{\text{2}}, \beta_{\text{3}}$ indicate the estimated parameters.

Abbreviations: SE.mean, standard error of the mean, SD standard deviation; CV, coefficient of variation; FC, full cohort; RS, random subcohort; CCI, case-cohort design with one-third proportion sample; CCII, case-cohort design with one-sixth proportion sample.
